# Draft Genome Sequence of *Lactococcus lactis* subsp. *lactis* bv. diacetylactis CRL264, a Citrate-Fermenting Strain

**DOI:** 10.1128/genomeA.01575-15

**Published:** 2016-02-04

**Authors:** Federico Zuljan, Martín Espariz, Victor S. Blancato, Luis Esteban, Sergio Alarcón, Christian Magni

**Affiliations:** a Laboratorio de Fisiología y Genética de Bacterias Lácticas, Instituto de Biología Molecular y Celular de Rosario (IBR-CONICET), Rosario, Argentina; b Instituto de Química de Rosario (IQUIR-CONICET), Rosario, Argentina; c Laboratorio Biotecnología e Inocuidad de los Alimentos, Facultad de Ciencias Bioquímicas y Farmacéuticas, Universidad Nacional de Rosario (UNR), Rosario, Argentina; d Facultad de Ciencias Médicas, UNR, Rosario, Argentina

## Abstract

We report the draft genome sequence of *Lactococcus lactis* subsp. *lactis* bv*.* diacetylactis CRL264, a natural strain isolated from artisanal cheese from northwest Argentina. *L. lactis* subsp. *lactis* bv*.* diacetylactis is one of the most important microorganisms used as starter culture around the world. The CRL264 strain constitutes a model microorganism in the studies on the generation of aroma compounds (diacetyl, acetoin, and 2,3-butanediol) by lactic acid bacteria. Our genome analysis shows similar genetic organization to other available genomes of *L. lactis* bv. diacetylactis strains.

## GENOME ANNOUNCEMENT

*Lactococcus lactis* is a mesophilic bacterium widely used in the production of fermented milk products, particularly cheese. Technological strains are divided into three phenotypes: *L. lactis*, *L. cremoris*, and *L. diacetylactis*. *L. lactis* IL1403 is a plasmid-free strain derived from *L. lactis* subsp. *lactis* bv*.* diacetylactis IL594 (*L. diacetylactis*) (1) and constitutes a model microorganism, being the first sequenced genome of a lactic acid bacterium (2).

The *L. diacetylactis* CRL264 strain was initially characterized for its capacity to ferment citrate (3), and it was later extensively characterized in our labs at the biochemical and genetic levels (4–11). Citrate is transported by a plasmid-encoded transporter (CitP) (4–6), whereas genes encoding the enzymes required for the intracellular metabolism of citrate are located in a large chromosomal operon (8). The citrate lyase enzyme complex is responsible for cleaving citrate into acetate and oxaloacetate, which is subsequently decarboxylated to pyruvate by the action of the soluble oxaloacetate decarboxylase (7, 9). Also, it has been demonstrated that acidification of the external medium is required for the induction of both transporter (plasmidic) and catabolic genes (chromosomal) (6–8, 10). Moreover, the route involved in the generation of the aroma compounds (*butBA* encoding 2,3-butanediol dehydrogenase and diacetyl-acetoin reductase, *als* encoding acetolactate synthetase, and *aldB* and *aldC* both encoding acetolactate decarboxylase) is also induced at low pH (10, 11).

In this report, the draft genome sequence of *L. diacetylactis* CRL264, consisting of 83 contigs, is presented. The genome sequence was determined by using an Illumina HiSeq 2000 platform (MR DNA, USA). Base-calling was carried out with HiSeq control software version 1.4.8. (Metrics sum, 2.60 Mbp; *N*_50_, 48.2 kbp; max, 252.7 kbp; min, 200 bp; G+C content, 36.4%; number of clusters, 3,891; number of reads, 7,782; size: 463.57 Mb; coverage, 424.68×. The short reads were *de novo* assembled with SeqMan NGen (DNASTAR Inc.). BLASTn analysis (all versus all) with the resulting contigs was performed, and those shorter than 1,000 bp and with an identity higher than 99% with sequences already contained in a longer contig were deleted. The remaining contigs were ordered and oriented with Advanced Pipmaker (12) and Mauve version 2.3.1 (13) using the annotated genome of *L. lactis* IL1403 as the genome of reference for *L. diacetylactis* CRL264.

The complete genome of strain CRL264 contains a total of 2,500 coding sequences; 63 structural RNAs were detected, and over 45% of the genes were assigned to specific subsystem categories by RAST (Rapid Annotations using Subsystem Technology) (14) and BASys (Bacterial Annotation System) (15). Manual curation of the genes was performed with the Seed viewer version 2.0, comparing the presence and absence of genes with closely related species. Comparative analysis of the chromosomal genome of the CRL264 strain among *L. diacetylactis* strains IL1403, LD61, TIFN2, and TIFN4 showed a clonal origin of the diacetylactis strain. Experimental and genomic comparative analysis will provide further insight into the strain’s capacity to produce diacetyl.

### Nucleotide sequence accession number.

The complete genome of *L. lactis* bv. diacetylactis CRL264 has been deposited at DDBJ/EMBL/GenBank under accession number LKPE00000000.
